# Lateral cephalometric diagnosis of asymmetry in Angle Class II subdivision compared to Class I and II

**DOI:** 10.1590/2176-9451.19.4.080-088.oar

**Published:** 2014

**Authors:** Aparecida Fernanda Meloti, Renata de Cássia Gonçalves, Ertty Silva, Lídia Parsekian Martins, Ary dos Santos-Pinto

**Affiliations:** 1 PhD in Orthodontics and Facial Orthopedics, School of Dentistry — State University of São Paulo (UNESP)/Araraquara.; 2 Specialist in Orthodontics and Facial Orthopedics, PUC-RJ.; 3 Adjunct professor, Department of Pediatric Dentistry and Orthodontics, School of Dentistry — State University of São Paulo (UNESP)/Araraquara.

**Keywords:** Facial asymmetry, Malocclusions, Radiography, Cephalometry

## Abstract

**Introduction:**

Lateral cephalometric radiographs are traditionally required for orthodontic treatment, yet rarely used to assess asymmetries.

**Objective:**

The objective of the present study was to use lateral cephalometric radiographs to identify existing skeletal and dentoalveolar morphological alterations in Class II subdivision and to compare them with the existing morphology in Class I and II relationship.

**Material and Methods:**

Ninety initial lateral cephalometric radiographs of male and female Brazilian children aged between 12 to 15 years old were randomly and proportionally divided into three groups: Group 1 (Class I), Group 2 (Class II) and Group 3 (Class II subdivision). Analysis of lateral cephalometric radiographs included angular measurements, horizontal linear measurements and two indexes of asymmetry that were prepared for this study.

**Results:**

In accordance with an Index of Dental Asymmetry (IDA), greater mandibular dental asymmetry was identified in Group 3. An Index of Mandibular Asymmetry (IMA) revealed less skeletal and dental mandibular asymmetry in Group 2, greater skeletal mandibular asymmetry in Group 1, and greater mandibular dental asymmetry in Group 3.

**Conclusion:**

Both IDA and IMA revealed greater mandibular dental asymmetry for Group 3 in comparison to Groups 1 and 2. These results are in accordance with those found by other diagnostic methods, showing that lateral cephalometric radiography is an acceptable method to identify existing skeletal and dentoalveolar morphological alterations in malocclusions.

## Abstract (pt)

**Introdução:**

as telerradiografias laterais são tradicionalmente solicitadas para planejamento ortodôntico, mas raramente utilizadas para avaliar assimetrias.

**Objetivo:**

o objetivo do presente estudo foi utilizar as telerradiografias laterais para identificar as alterações morfológicas esqueléticas e dentoalveolares existentes na má oclusão de Classe II subdivisão e compará-las com a morfologia existente nas más oclusões de Classe I e II.

**Métodos:**

noventa telerradiografias laterais iniciais de adolescentes brasileiros de ambos os sexos, com idade cronológica entre 12 e 15 anos, foram divididas em três grupos randomizados e proporcionais: Grupo 1 (Classe I), Grupo 2 (Classe II) e Grupo 3 (Classe II subdivisão). A análise das telerradiografias laterais envolveu mensurações angulares, mensurações lineares horizontais e dois índices de assimetria, estipulados para o presente estudo.

**Resultados:**

foi identificada, de acordo com o Índice de assimetria dentária (IAD), uma maior assimetria dentária inferior no Grupo 3. O Índice de assimetria mandibular (IAM) revelou menor assimetria esquelética e dentária no Grupo 2, maior assimetria esquelética no Grupo 1 e maior assimetria dentária inferior no Grupo 3.

**Conclusão:**

o IAD e o IAM mostraram maior assimetria dentária inferior no Grupo 3 do que nos Grupos 1 e 2. Esses resultados estão de acordo com os encontrados em outros métodos de diagnóstico, indicando que a telerradiografia lateral é um método aceitável para avaliar alterações morfológicas esqueléticas e dentoalveolares nas más oclusões.

## INTRODUCTION

Class II subdivision is characterized by an asymmetrical posterior occlusal relationship in which the dental arches demonstrate a Class I relationship on one side and a Class II relationship on the other side. This asymmetrical occlusal relationship is of skeletal and/or dentoalveolar origin.^1^ Knowing the origin of this asymmetry is extremely important to ensure correct treatment of individuals with Class II subdivision.

Slight degrees of facial asymmetry are common among the general population.^2^ Individuals with Class II subdivision typically present an accentuated degree of asymmetry that involves the lower third of the face and the mandible.^3,4,5^

Alavi et al^6^ reported the distal position of the first lower molar as the main cause of Class II subdivision asymmetry. Additionally, these authors stated that asymmetry could have dentoalveolar or skeletal etiology. Rose et al^7^ also observed first lower molar in Class II subdivision asymmetry positioned more posteriorly on the Class II side; however, these authors stated that asymmetry resulted from dentoalveolar involvement without observable changes in the jaw. The position of dental midlines in relation to the facial midline was examined and revealed^8^ that lower dental midline deviation was more common than upper midline deviation, suggesting the cause of this asymmetry to be mandibular in nature.

While observing maxillary and mandibular changes in Class II subdivision and Class I malocclusions, Janson et al^9^ showed that dentoalveolar changes occurred in jaws without positional asymmetry. The main cause of Class II subdivision relationship was the distal position of lower molars on the Class II side. The position of upper mesial molars, also on the Class II side, was a secondary cause. The lower dental midline also presented more frequent deviations on the Class II side than the upper dental midline did. Therefore, this study^9^ as well as others^4,7,8,10,11^ demonstrated that asymmetries present in Class II subdivision patients are mainly of dentoalveolar origin.

Computed tomography is considered an optimal diagnostic method for asymmetry assessment,^12^ but the cost of this method is higher and its radiation dose is greater in comparison to other methods. Photographs have been compared to posteroanterior radiographs, but no significant correlation has been found between methods.^13^ Edler et al^14^ argued that photographs should be used simultaneously with posteroanterior radiographs. When photographs were compared to submentovertex radiographs and posteroanterior radiographs,^15^ a small correlation was found between methods. Posteroanterior radiographs allow observations of vertical and transversal changes; however, reports in the literature^6-9,11,12^ have noted a greater change in the anteroposterior positioning of molars in Class II subdivision malocclusion.

Although anteroposterior changes can be observed with submentovertex radiographs, Lew and Tay^16^ found a distortion in linear measurements taken with these radiographs. Additionally, Arnold et al^17^ reported difficulties in using submentovertex radiographs. The use of 45° cephalometric radiographs offers another method that allows visualization of structures in the anteroposterior direction, but this method is not routinely applied because it requires two further radiographic images in addition to those required for basic orthodontic documentation. Study models may be used for observation of dental structures in the anteroposterior direction, but these models do not allow skeletal observations. In addition, panoramic radiographs do not enable anteroposterior morphological alterations to be visualized.^18,19^

Because they are traditionally required for orthodontic treatment, lateral cephalometric radiographs allow visualization of anteroposterior structures in a simple manner without additional costs to the orthodontist. Only one study^6^ has used lateral cephalometric radiographs to observe the position of molars and the existence of an asymmetrical mandibular relationship in the anteroposterior direction.

Therefore, the objectives of our study were to use lateral cephalometric radiographs to identify skeletal and dentoalveolar morphological alterations in cases of Class II subdivision; to compare these changes with morphology of Class I and Class II; and to assess the incidences of dental and skeletal symmetry and asymmetry of the maxilla and mandible.

## MATERIAL AND METHODS

This research was approved by the School of Dentistry - State University of São Paulo Institutional Review Board. The sample comprised 90 male and female Brazilian children aged between 12 and 15 years old, randomly selected in the archives of the School of Dentistry, State University of São Paulo/Araraquara. The sample was divided into three equal groups of Class I, Class II or Class II subdivision patients. Malocclusion criteria were based on the occlusal relationship between upper and lower arches obtained on study models and photographic documentation. Molar and canine relationships in Group 1 (Class I) were bilateral and symmetrical, whereas those in Group 2 (Class II) were displaced in more than half the width of a cusp. In Group 3 (Class II subdivision), molar and canine exhibited a Class I relationship on one side and a Class II relationship on the other side. Additional inclusion criteria for all groups were as follows: normal lower arch or a lower arch with slight lower-anterior crowding, and the presence of all permanent teeth in the dental arches (from first molar to first molar) with eminent eruption or eruption of second molars. Subjects were excluded if they had occlusal interferences that might cause functional alterations (e.g., dental crossbite, open bite or history of facial trauma).

Standardized lateral cephalometric radiographs were taken with patients' teeth in maximum habitual intercuspation with relaxed lips and face positioned with Camper's plane parallel to the ground. Radiographs were taken with Rotograph plus model MR05, adjusted for 85 Kvp, 10 mA and 0.5 seconds of exposure time. The equipment had fixed and constant focus-object distance of 1.5 meters. The chassis with Kodak^tm^ - TMG/RA, 20.3cm x 25.4 cm film was positioned 15 cm away from the medial sagittal plan, giving an average magnification factor of 10%.

**Figure 1:**
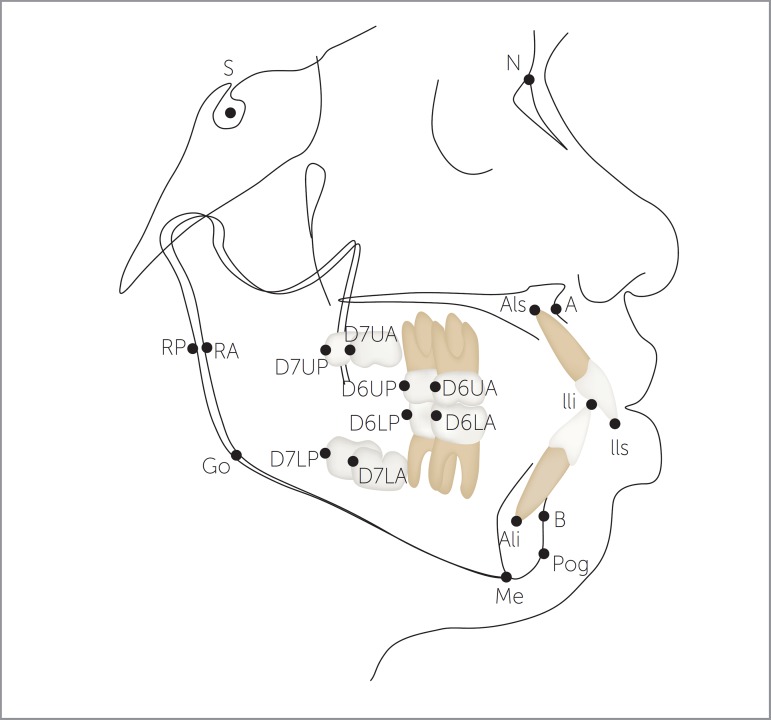
Skeletal and dental cephalometric points. S (Sella), N (Nasion), **A** (Subspinal), **B** (Supramental), **Go** (Gonial), **Me** (Mentalis), **Pog** (Pogonion), **IIs** (Incisal edge of maxillary central incisor), **AIs** (Apex of upper incisor), **IIi** (Incisal edge of the lower central incisor), **AIi** (Apex of lower incisor), **RA** (Anterior ramus), **RP** (Posterior ramus), **D7UA** (Point in the distal face of the most anterior image of the second upper molar crown), **D7UP** (Point in the distal face of the most posterior image of the second upper molar crown), **D6UA** (Point in the distal face of the most anterior image of the first upper molar crown), **D6UP** (Point in the distal face of the most posterior image of the first upper molar crown), **D7LA** (Point in the distal face of the most anterior image of the second lower molar crown), **D7LP** (Point in the distal face of the most posterior image of the second lower molar crown), **D6LA** (Point in the distal face of the most anterior image of the first lower molar crown), **D6LP** (Point in the distal face of the most posterior image of first lower molar crown).

Cephalometric analysis was performed by digitizing twenty-one points identified in the lateral radiographs (Fig 1) by the same researcher using a Numonics AccuGrid digitizer (TPL 1212 - Kurta, Seymour, Connecticut - USA) and Dentofacial Planner Plus, version 6.5, 1995 (Dentofacial software Inc. Toronto, Ontario - Canada). Radiographs were randomly digitized by means of simple random sampling without group identification.

**Figure 2:**
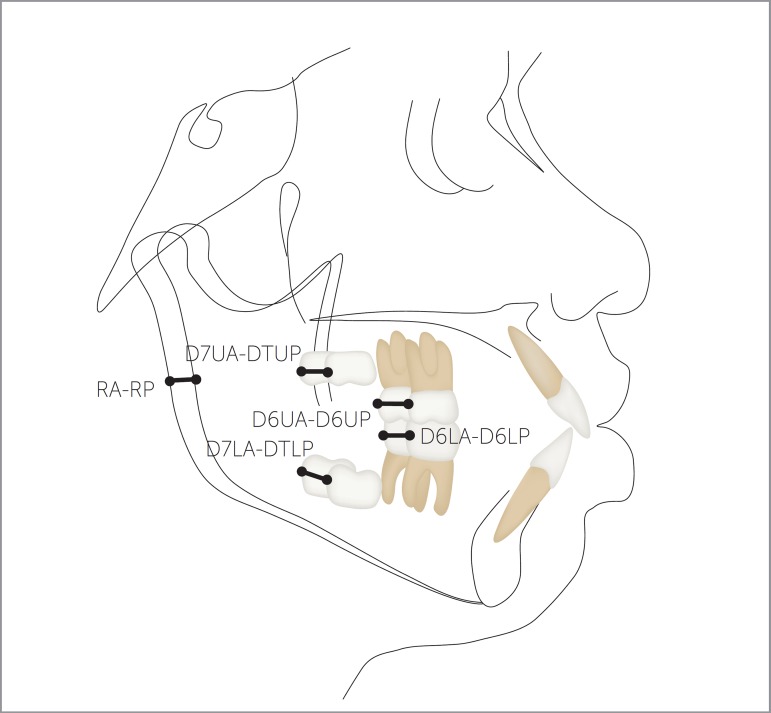
Skeletal and dental linear cephalometric measurements. **RA-RP** (Horizontal distance between the anterior (RA) and posterior (RP) images of the posterior mandibular borders), **D7UA-D7UP** (Horizontal distance between the D7UA and D7UP points), **D6UA-D6UP** (Horizontal distance between the D6UA and D6UP points), **D7LA-D7LP** (Horizontal distance between the D7LA and D7LP points), **D6LA-D6LP** (Horizontal distance between the D6LA and D6LP points).

For characterization of the sample, the following angular measurements were used: SNA, SNB, ANB, SNPP (angle formed by the SN line and the palatal plane [ANS - PNS]), SNOP (angle formed by the SN line and the occlusal plane [Op - Oa]), SNGoMe, U1.SN, L1GoMe, U1.L1 and NAPog. Study analysis involved two indexes (i.e., the index of dental asymmetry and the index of mandibular asymmetry) as well as five linear measurements (RA-RP, D7UA-D7UP, D6UA-D6UP, D7LA-D7LP and D6LA-D6LP) (Fig 2).

### Index of dental asymmetry (IDA)

An IDA was developed based on the difference in distance between the most anterior and the most posterior molars in the upper and lower dental arches [IDA1 = (D6UA-D6UP) - (D6LA-D6LP)]. Similarly, this index was applied for second upper and lower molars [IDA2= (D7UA-D7UP) - (D7LA-D7LP)].

Mathematically, a difference of zero represents upper-lower dental symmetry. A variation from normality of ± 0.5 mm was used for the tolerance criterion; this value corresponds to the degree of magnification between the right and left sides in cephalometric measurements. Values greater than 0.5 mm represented a greater distance between upper molars than between lower molars, and thus indicated upper dental asymmetry. Values of less than -0.5 mm represented a greater distance between lower molars than between upper molars, and thus indicated lower dental asymmetry.

For example, the IDA using the first molars is described as follows:

» IDA1= (D6UA-D6UP) - (D6LA-D6LP), where

» (D6UA-D6UP) = distance between the most anterior image of the upper first molar (D6UA) and the most posterior molar (D6UP); and

» (D6LA-D6LP) = distance between the most anterior image of the lower first molar (D6LA) and the most posterior molar (D6LP).

If:

» IDA > 0.5 mm = upper dental asymmetry;

» IDA < -0.5 mm = lower dental asymmetry;

» -0.5 mm ≥ IDA ≤ 0.5 mm = upper and lower dental symmetry.

### Index of mandibular asymmetry (IMA)

Following the same logic, an IMA was developed based on the difference in distance between the most anterior and the most posterior portions of the mandibular ramus, and the distance between the most anterior and the most posterior lower first molars [IMA1 = (RA-RP) - (D6LA-D6LP)]. Similarly, this index was applied for second molars [IMA2= (RA-RP) - (D7LA-D7LP)].

Mathematically, a difference of zero between skeletal and dental mandibular distances indicated dental and skeletal mandibular symmetry. As above, a tolerance criterion of ± 0.5 mm was used to indicate variation from normality. Values greater than 0.5 mm represented skeletal asymmetry, as the anterior-posterior extent of the ramus was greater than that of the lower molars. On the other hand, values of less than -0.5 mm represented dental asymmetry, as the anterior-posterior extent of the lower molars was greater than that of the mandibular ramus.

For example, the IMA using the first molars was described as follows:

» IMA1= (RA-RP) - (D6LA-D6LP), where

» (RA-RP) = distance between the most anterior image of the mandibular ramus (RA) and the most posterior one (RP); and

» (D6LA-D6LP) = distance between the most anterior image of the lower first molar (D6LA) and the most posterior one (D6LP).

If:

» IMA > 0.5 = mandibular skeletal asymmetry;

» IMA < -0.5 = mandibular dental asymmetry;

» -0.5 mm ≥ IMA ≤ 0.5 mm = skeletal and dental mandibular symmetry.

### Statistical analysis

To assess consistency of measurements, six radiographs from each group were digitized twice by the same researcher with an interval of two weeks in between. The intra-class correlation coefficient (ICC) was used to assess reliability of the variable measurement process. Measurements were considered adequate when the ICC value was greater than 0.95.

To test the hypothesis that mean angular measurements were equivalent for the three groups, an analysis of variance (ANOVA) was used. When Levene's prior test rejected the hypothesis of homogeneity of variances, Brown-Forsythe test was used to verify equality of means. Scheffé's multiple comparison test was used to detect significant differences between groups.

A chi-square test was used to test the hypothesis that the proportion of subjects with asymmetries did not differ between groups, and to determine whether there was an association between category of asymmetry and group. A 95% confidence level (p < 0.05) was considered statistically significant. Statistical analyses were performed using SPSS software, version 16.0 for Windows (release 16.01 - Nov. 2007; SPSS Inc., 1989-2007).

## RESULTS

Reliability of the method was satisfactory; ICC values for replicate measurements were greater than 0.99 for angular measurements and greater than 0.96 for linear measurements. The calculated ICC value was greater than 0.98 for all variables.

The analysis of differences between groups (Table 1) confirmed greater mandibular retrusion (smallest SNB) and greater lower incisor inclination (greater L1.GoMe) for Group 2 in comparison to the other groups (1 and 3). Group 1 had smaller maxillomandibular differences (smallest ANB) and lower facial convexity (smallest NAPog) than the other groups (2 and 3). Despite significant ANOVA result for the U1.L1 measurement, Scheffé's multiple comparison test was unable to detect significant differences between groups.

**Table 1:** Mean and standard deviation of measurements and analysis of variance (ANOVA) to test the hypothesis that the means of the three groups are the same

Cephalometric measurement	Group 1 Class I	Group 2 Class II	Group 3 Class II Subdivision	p
	Mean ±SD	Mean ±SD	Mean ±SD	
**Characterization of the groups**				
SNA	82.19± 3.54	81.80 ± 2.73	83.43± 4.56	0.208
SNB	79.81^a^ ± 3.23	76.04^b^ ± 2.82	78.97^a^ ± 4.33	**0.000**
ANB	2.37^a^ ± 2.03	5.77^b^ ± 2.07	4.46^b^ ± 2.05	**0.000**
SN.PP	7.63 ± 3.14	8.22 ± 3.58	7.62 ± 3.85	0.756
SN.OP	19.95 ± 2.74	19.47 ± 3.59	19.63 ± 4.68	0.884
SN.GoMe	34.99 ± 5.21	34.55 ± 4.17	32.16 ± 5.72	0.073
U1.SN	106.20± 7.88	106.81 ± 6.74	104.93 ± 7.14	0.597
L1.GoMe	91.49^a^ ± 8.60	97.50^b^ ± 6.12	95.49^ab^ ± 6.35	**0.005**
U1.L1	127.33 ± 12.90	121.15 ± 8.52	127.41 ± 9.84	**0.036**
NAPog	3.62^a^ ± 5.12	9.30^b^ ± 5.62	7.11^b^ ± 4.91	**0.000**
**Skeletal and dental linear **				
RA-RP(1)	1.38 ± 0.88	1.38 ± 1.60	1.37 ± 1.22	1.000
D6UA-D6UP	1.26^a^ ± 0.79	1.60^ab^ ± 1.19	2.02^b^ ± 1.23	**0.028**
D7UA-D7UP	1.27^a^ ± 0.82	1.57^ab^ ± 1.15	1.96^b^ ± 1.16	**0.045**
D6LA-D6LP(1)	1.20^a^ ± 0.80	1.87^b^ ± 1.11	2.51^b^ ± 1.78	**0.001**
D7LA-D7LP(1)	1.15^a^ ± 0.75	1.81^b^ ± 1.10	2.48^b^ ± 1.81	**0.001**

(1) Brown-Forsythe statistics (Levene's test rejected the hypothesis of homogeneity of variance).

As shown in Table 1, the RA-RP distance was similar for all groups. Therefore, if image distortions or variations in head position occurred, they were similar for all groups. In contrast, the dental measurements differed significantly among groups. Differences in distance between first upper molars (D6UA-D6UP) and second upper molars (D7UA-D7UP) were smaller in Group 1 than Group 3; yet the values for these groups did not differ from those of Group 2. Distances between first lower molars (D6LA-D6LP) and second lower molars (D7LA-D7LP) were smaller in Group 1 than Groups 2 or 3. All dental measurements were greater in Group 3 than in Groups 1 or 2.

The proportion of subjects with skeletal and dental mandibular symmetry, skeletal mandibular asymmetry and/or dental mandibular asymmetry was determined in the three groups by means of the IMA using first (IMA1) or second (IMA2) molars as reference. Despite the greater proportion of subjects with skeletal asymmetry in Group 1, the greater proportion of subjects with skeletal and dental symmetry in Group 2 and the greater proportion of subjects with dental asymmetry in Group 3, the chi-square test revealed no significant association between asymmetry and group in IMA1 (Table 2). Additionally, there was no statistically significant difference among the means of IMA1 for each asymmetry category. However, a greater incidence of dental mandibular asymmetry was observed in Group 3 than in Groups 1 or 2, and a greater incidence of skeletal asymmetry was observed in Group 2 than in Groups 1 or 3 (Table 3). When the second molar was used to calculate the IMA2, there was no significant association between asymmetry and group membership (Table 2). Finally, the magnitude of dental mandibular asymmetry in Group 1 was smaller than that in Groups 2 or 3 (Table 3).

**Table 2:** Number and proportion of individuals according to group and category of the index of asymmetry and results of chi-square test for the association between asymmetry and group

Index / Category of asymmetry	Group 1		Group 2		Group 3	
	n	%	n	%	n	%
	**IMA1 ( χ²= 8.66; df=4; p=0.070)**					
Dental asymmetry	10	33.3	14	46.7	20	66.7
Symmetry	7	23.3	9	30	4	13.3
Skeletal asymmetry	13	43.3	7	23.3	6	20
Total	30	100	30	100	30	100
	**IMA2 (χ²= 9.15; df=4; p=0.057)**					
Dental asymmetry	11	36.7	13	43.3	20	66.7
Symmetry	6	20.0	10	33.3	4	13.3
Skeletal asymmetry	13	43.3	7	23.3	6	20.0
Total	30	100.0	30	100.0	30	100.0
	**IDA1 (χ² = 16.33; df=4; p=0.003)**					
Dental asymmetry	3	10.0	8	26.7	13	43.3
Symmetry	23	76.7	18	60.0	8	26.7
Skeletal asymmetry	4	13.3	4	13.3	9	30.0
Total	30	100.0	30	100.0	30	100.0
	**IDA2 (χ²= 14.60; df=4; p=0.006)**					
Dental asymmetry	3	10.0	7	23.3	12	40.0
Symmetry	23	76.7	19	63.3	9	30.0
Skeletal asymmetry	4	13.3	4	13.3	9	30.0
Total	30	100.0	30	100.0	30	100.0

IMA = index of mandibular asymmetry; IMA1 = (RA-RP) - (D6LA - D6LP); IMA 2 = (RA-RP) - (D7LA - D7LP).

IDA = index of dental asymmetry; IDA1 = (D6UA-D6UP)-(D6LA-D6LP); IDA 2 = (D7UA-D7UP)-(D7LA-D7LP).

**Table 3:** Mean and standard deviation of the index of asymmetry and results of the analysis of variance (ANOVA) to test the hypothesis of equality of the means of the three groups, according to the category of asymmetry.

Index / Category of asymmetry	Group 1 Mean ± SD	Group 2 Mean ± SD	Group 3 Mean ± SD	p
**IMA1 (RA-RP) – (D6LA – D6LP)**				
Dental asymmetry	-1.35 ± 0.54	-1.89 ± 0.85	-2.23 ± 1.12	0.064
Symmetry	-0.21 ± 0.42	-0.04 ± 0.28	0.25 ± 0.29	0.120
Skeletal asymmetry	1.57 ± 0.68	1.74 ± 0.57	1.57 ± 0.73	0.840
**IMA2 (RA-RP) – (D7LA – D7LP)**				
Dental asymmetry	-1.18^a^ ± 0.47	-1.97^b^ ± 0.8	-2.16^b^ ± 1.22	**0.031**
Symmetry	-0.12 ± 0.44	-0.02 ± 0.31	0.18 ± 0.21	0.429
Skeletal asymmetry	1.58 ± 0.67	1.83 ± 0.78	1.55 ± 0.73	0.721
**IDA1 (D6UA – D6UP) – (D6LA – D6LP))**				
Dental asymmetry	-0.80^a^ ± 0.20	-1.28^a^ ± 0.24	-2.52^b^ ± 0.40	0.002
Symmetry	-0.01 ± 0.57	-0.08 ± 0.27	0.18 ± 0.16	0.050
Skeletal asymmetry	1.13 ± 1.03	0.90 ± 0.16	1.86 ± 0.84	0.058
**IDA2 (D7UA-D7UP)–(D7LA-D7LP)**				
Dental asymmetry	-0.73^a^ ± 0.23	-1.31^a^ ± 0.39	-2.70^b^ ± 1.00	**0.001**
Symmetry	0.07^ab^ ± 0.26	-0.05^a^ ± 0.25	0.21^b^ ± 0.19	**0.031**
Skeletal asymmetry	1.05 ± 0.47	0.75 ± 0.17	1.66 ± 0.82	0.082

The proportion of subjects with dental symmetry, upper dental asymmetry and/or lower dental asymmetry was determined in the three groups by IDA1 and IDA2. A chi-square test revealed significant association between asymmetry and group membership. The proportion of individuals with dental symmetry was significantly greater in Groups 1 and 2 than in Group 3. In Group 3, there was a high frequency of lower dental asymmetry (Table 2). The magnitude of lower dental asymmetry was also greater in Group 3 than in Groups 1 or 2 (Table 3).

## DISCUSSION

In this study, lateral radiographs were used to assess the nature of asymmetries in individuals with Class II subdivision (Group 3) compared to control groups of individuals with bilateral symmetric Class I (Group 1) or bilateral symmetric Class II (Group 2) relationship. Although other diagnostic methods are more frequently used than lateral radiography, these methods are accompanied by specific disadvantages.^6-9,11-17^

Lateral cephalometric radiographs allow anteroposterior structures to be visualized in a simple manner without additional costs to the orthodontist, as they are traditionally required for diagnostic and treatment planning. However, as other radiographic methods, errors in head positioning may occur.^20^ The head may rotate along transverse, anteroposterior, or vertical axes. Rotations along the transverse axis do not cause image distortions because the head remains parallel to the X-ray source. Rotation produces relative changes in the location of images on the film, but none in the relationships of structures that could cause errors in the process of radiographic measurement. Rotation along the anteroposterior axis affects vertical measurements. Although bilateral structures move equally, vertical measurements increase or decrease based on the direction of rotation. Rotation along the vertical axis could influence horizontal measurements, as analyzed in this study.^20^ When the head rotates along the vertical axis, the length of the mandibular body gradually decreases as the rotation angle increases along the direction of the film. Alteration in length is typically approximately 1%; however, this percentage may increase to -5.78% when the angle of head rotation varies between -5 and -15 degrees.^20^ The effects of head rotation on measurements of mandible and molars are equal in magnitude. Therefore, the absolute but not relative distance between these structures is affected, as demonstrated by the indexes of asymmetry of this current study.

According to Kjellberg et al,^19^ radiographic extent, head position and distortions can be ignored when an index is used to calculate linear measurements. Habets et al^20^ also believe that morphological differences of size, calculation and interpretation of findings can be excluded by certain indexes such as those used in the current study.

Our sample showed a few cephalometric differences related to the characteristics of malocclusion. For example, individuals in Group 1 presented smaller ANB and less facial convexity. These differences reflect the characteristics of the groups, confirming that individuals in Group 2 presented greater mandibular retrusion than those in Groups 1 and 3. Although Group 3 has a Class II relationship on one side, the Class I relationship on the other side produces smaller retrusion than in individuals with bilateral Class II (Group 2). Azevedo et al^4^ reported that skeletal involvement in individuals with Class II subdivision is typically small. Greater buccal positioning of lower incisors in individuals with Class II arises due to dentoalveolar compensation for their greater mandibular retrusion, which results in a significantly more closed interincisal angle.

Distances between first and second upper or lower molars (Table 2) were always smaller in Group 1 than in Groups 2 and 3, thus revealing that this type of relationship is associated with greater dental symmetry. IDA1 and IDA2 identified greater dental symmetry in Groups 1 and 2, indicating great concordance between our direct measurements and the results of these indexes.

Similarly, IMA1 and IMA2 revealed greater skeletal mandibular asymmetry in Group 1 (Table 2). This result is supported by the findings by Sezgin et al^21^ who found greater asymmetry in individuals with Class I than those with normal occlusion. They also found^22^ asymmetry in Class I patients, with the mandible less anterior and highly positioned in hyperdivergent patients than in hypodivergent.

IMA revealed greater skeletal and dental mandibular symmetry in Group 2 than in Groups 1 and 3. Although Group 2 tended to show greater symmetry than individuals in the other groups, their skeletal asymmetry (when present) was greater in magnitude than that of Groups 1 and 3.

IMA revealed greater dental mandibular asymmetry in Group 3 than those in Groups 1 and 2. IDA also showed individuals in Group 3 to have greater lower dental asymmetry than Groups 1 and 2. These results corroborate those presented by authors^4,7-11^ using other diagnostic methods, such as posteroanterior radiography, submentovertex radiography, 45° radiography, study models and photographs. Alavi et al^6^ used lateral radiograph to investigate asymmetries in individuals with Class II subdivision. Nevertheless, the authors were not able to determine whether these changes arose due to dentoalveolar or skeletal etiology.

## CONCLUSION

» Two indexes of asymmetry and direct measurements were presented as part of a new evaluation method used to identify dental and skeletal asymmetries by means of lateral cephalometric radiography.
» Distances between first and second upper or lower molars were always less in the Class I group and greater in the Class II subdivision group, in accordance with new IDA indexes which identified greater dental asymmetry in individuals with Class II subdivision than those with Class I and Class II.
» New IMA indexes revealed less skeletal and dental mandibular asymmetry in individuals with Class II, and greater skeletal mandibular asymmetry in individuals with Class I.
» IMA and IDA suggested that Class II subdivision individuals had greater mandibular dental asymmetry than Class I or Class II.
